# Inequities in Children’s Food Environment: A Comparison between Slum and Non-slum Settings in Urban Bangladesh

**DOI:** 10.1007/s11524-026-01144-5

**Published:** 2026-08-31

**Authors:** Tania Ahmed Chowdhury, Nasrin Akhter, Kaniz Fatima Emy, Akhi Akter, Ishrat Hossain, Rezwana Jahan, Mohammad Jahangir Alam, Pepijn Schreinemachers, Ee Von Goh

**Affiliations:** 1 Radda Maternal Child Health - Family Planning (Radda MCH-FP) Centre, Dhaka, Bangladesh; 2 Moulovibari Research and Partnership Hub (MoRPH), Dhaka, Bangladesh; 3 World Vegetable Center, Bangkok, Thailand; 4 World Vegetable Center, Tainan, Taiwan

**Keywords:** Urban child nutrition, Dietary diversity, Slum health disparities, Food environment, Food affordability, Nutrition policy

## Abstract

Children living in urban slums face persistent nutritional and health inequities, many of which are rooted in the food environment. This study examined the drivers of children’s food behaviors in Dhaka, Bangladesh, comparing slum and non-slum settings through a cross-sectional survey of 300 mothers of school-aged children (8–12 years) and focus group discussions. Quantitative analysis revealed disparities in diet quality: children in non-slum areas had a 13% higher dietary quality score (*p* < 0.001) and consumed 57% more servings of fruit and vegetables (*p* < 0.001) than their peers in slum areas. Household income, maternal education, and monthly spending on fruits and vegetables were significant predictors of fruit and vegetable intake and dietary quality scores. Qualitative insights further showed that mothers in slum areas prioritized giving their children energy-dense, animal-source foods. In contrast, mothers in non-slum areas emphasized dietary diversity, including both animal- and plant-based foods, and tried to limit their children’s unhealthy food choices. Differences in food marketing exposure and inadequate school food environments further reinforced the observed differences in dietary behavior. Addressing these food inequities requires integrated urban nutrition strategies, including affordable access to fresh produce, regulation of food marketing to children, and strengthened school nutrition programs.

**Supplementary Information:**

The online version contains supplementary material available at https://doi.org/10.1007/s11524-026-01144-5.

## Introduction

By 2050, more than two-thirds of the global population is projected to reside in urban areas [1]. Rapid urbanization is transforming food environments worldwide, reshaping dietary patterns and exacerbating socioeconomic inequities [2]. Vulnerable populations, including children living in informal settlements (slums), are disproportionately affected by limited access to affordable and nutritious foods [3].

Dhaka, one of the world’s fastest-growing megacities, exemplifies these challenges. The city has grown 3.1% annually from 2010 to 2020 [4]. The city faces stark inequalities between the slum and non-slum populations. Approximately one-third of Dhaka’s 20 million residents live in slums, and children account for around 40% of this population [5]. Evidence from Africa and Latin America suggests that children in slum settings often consume fewer fruits and vegetables, have lower dietary diversity, and are more exposed to unhealthy food options than their non-slum peers [6–8]. Similar disparities are likely to exist in Dhaka, but evidence documenting them is currently lacking.

Food insecurity is a major feature of urban poverty in Dhaka and may be an important pathway linking slum residence to poor dietary quality. Recent evidence from Dhaka found that during periods of high food inflation, only 21% of slum households remained food secure, while the remainder experienced mild (46%), moderate (21%), or severe (12%) food insecurity [9]. Food-insecure households commonly coped by reducing food consumption, switching to less nutritious foods, borrowing money, or selling assets, all of which may adversely affect children’s dietary diversity and fruit and vegetable consumption.

Adequate consumption of fruits and vegetables is essential for healthy growth and development and plays a critical role in reducing the risk of both undernutrition and non-communicable diseases [10]. Yet children in low-income urban areas frequently face food environments where fresh produce is unaffordable, inaccessible, or culturally less desirable compared to cheaper, processed alternatives [11, 12]. These inequities are compounded by inadequate school food provision, aggressive marketing of ultraprocessed foods, and structural barriers that reinforce dietary gaps [13].

Childhood, the period of life between infancy and adolescence, is a crucial life stage for establishing dietary habits. During this time, individuals experience rapid growth—gaining approximately 20% of their adult height and 50% of their adult weight—alongside cognitive and emotional development [14]. Children are particularly vulnerable to food system failures during this period [15]. Recognizing this, UNICEF’s Nutrition Strategy 2020–2030 emphasizes that every child has the right to safe, affordable, and nutritious diets [16].

In this study, children’s diets are conceptualized as being shaped by the interaction between food environments and household socioeconomic circumstances. Drawing on established frameworks in the food environment literature, the food environment is understood to encompass both external factors—such as the availability, accessibility, and affordability of foods in the home, school, and community—and personal factors, including caregiver feeding practices, nutrition knowledge, and exposure to food marketing [17, 18]. These dimensions do not operate in isolation: they interact with household-level characteristics to shape what children eat.

Household socioeconomic circumstances—including income, housing conditions, parental education, and maternal employment—may influence children’s diets through distinct but overlapping pathways. Income determines purchasing power and dietary diversity, parental education shapes nutrition knowledge and feeding practices, and maternal employment influences both resources and time for food preparation. Housing conditions further affect diet through storage capacity, sanitation, and cooking facilities. Evidence from LMICs consistently shows that higher income and maternal education are associated with improved dietary diversity and fruit and vegetable intake in children [19–21]. In addition, caregiver food literacy and social support have been identified as important determinants of child dietary quality [22].

Despite this body of evidence, studies in South Asia have rarely examined how these socioeconomic relationships with diet quality differ between children living in slum and non-slum settings. Where slum and non-slum comparisons do exist, they tend to focus on anthropometric outcomes such as height, weight, and anemia rather than dietary diversity or food group consumption per se [23, 24]. While global evidence has documented how food environments influence children’s diets, few studies in South Asia explicitly compare dietary diversity and fruit and vegetable consumption among children in slum and non-slum areas [25].

Accordingly, this study examines how children in Dhaka interact with their food environments and the socioeconomic and cultural factors shaping their diets. Specifically, it (a) compares dietary diversity and fruit and vegetable consumption of children in slum and non-slum areas; (b) assesses how socioeconomic factors influence diet quality; and (c) contextualizes these disparities using qualitative insights from mothers on healthy diet perceptions, affordability, and prioritization. Together, these findings provide new evidence on the socioeconomic, behavioral, and cognitive dimensions of urban nutritional inequity in Dhaka and offer policy insights relevant to other rapidly urbanizing cities in South Asia and beyond.

## Data and Methods

### Study Area and Design

Data were collected in Mirpur, Dhaka North City Corporation—a 59 km^2^mixed urban area with about 633,000 residents [26], where formal and informal housing areas coexist, creating sharp contrasts in public services and food access. In Bangladesh, “slums” are defined in the national census as clusters of ≥ 10 households with inadequate housing, high density, poor sanitation, and low socioeconomic conditions [27, 28]. The term “slum” is used throughout this study for consistency with local definitions.

The study combined quantitative and qualitative approaches to examine factors influencing children’s dietary patterns and food environments. A household survey assessed children’s dietary diversity, fruit and vegetable intake, household socioeconomic characteristics, and food environment factors. Focus group discussions (FGDs) with mothers/caregivers explored perceptions, affordability constraints, and household practices influencing children’s food choices.

Ethical approval was obtained from the Public Health Foundation Bangladesh (PHFBD-ERC-FP02/2024) and the World Vegetable Center Institutional Biosafety and Research Ethics Committee (IBREC-2024-003). Informed consent and child assent were secured, with strict confidentiality maintained.

### Quantitative Data Collection

The study population consisted of school-aged children aged 8–12 years, with their mothers/caregivers serving as the primary respondents. A multistage sampling approach was used. Four wards were randomly selected from Dhaka North City Corporation (DNCC). Within each selected ward, one school serving either a slum or non-slum community was purposively selected. This resulted in four participating schools (two slum and two non-slum schools).

The sample size was calculated using the formula *n* = *z*^2^*pq*/*d*^2^, where *z* = 1.96 (95% confidence level), *p* = 0.25 (estimated proportion of children consuming recommended vegetable intake), *q* = 1 − *p* = 0.75, and* d* = 0.05 (margin of error). The calculated sample size was 288 respondents. Considering potential non-response and incomplete data, additional mothers/caregivers were approached. From each school, mothers/caregivers of children aged 8–12 years were randomly selected from class lists using Excel-generated random numbers. After excluding incomplete questionnaires and records with missing key variables, 300 mothers/caregivers were included in the final analysis, representing 300 school-aged children.

A semi-structured questionnaire was administered to collect household-, caregiver-, child-, and food environment-related information. Modules included household composition, housing characteristics, kitchen and sanitation facilities, food access and availability, food expenditure, and affordability indicators. The food environment assessment included market access, food purchasing frequency, availability of foods consumed in the previous week, and perceived affordability of fruits and vegetables.

Dietary outcomes were assessed using the Diet Quality Questionnaire (DQQ) and 24-h dietary recall approach [29, 30], together with a semi-quantitative food frequency questionnaire (SQFFQ) previously developed for the local population [31]. The main dietary outcomes included Dietary Diversity Score (DDS), fruit and vegetable intake, NCD-Protect score, and NCD-Risk score.

The DQQ is a standardized dietary assessment tool designed to capture food group consumption and diet quality indicators. The DQQ was implemented following official guidance, with food items adapted to the local food context. Dietary information was collected from mothers/caregivers regarding foods consumed by their child during the previous 24 h. The DDS was calculated based on consumption of the ten defined DQQ food groups. A score of one was assigned when a child consumed any food item within a food group and zero when no food from that group was consumed. The total DDS ranged from 0 to 10, with higher scores indicating consumption of a greater number of food groups.

Weekly fruit and vegetable intake was assessed using the adapted SQFFQ. The fruit and vegetable sections were expanded to include commonly consumed local varieties identified through consultation with nutritionists and consideration of the local food context. Mothers/caregivers reported the number of days per week and frequency per day that each food item was consumed by the child. Portion sizes were estimated using commonly used household measures (e.g., bowls, spoons, and pieces). Standardized portion weights were developed with guidance from nutritionists, and weekly servings were calculated by combining reported frequency and estimated portion size.

NCD-Protect and NCD-Risk scores were calculated based on DQQ responses following the Global Diet Quality Project indicators [29, 30]. The NCD-Protect score reflects consumption of food groups associated with dietary factors protective against non-communicable diseases, including fruits, vegetables, whole grains, pulses, nuts, and seeds. One point was assigned for consumption of each protective food group during the previous 24 h, and points were summed to generate the composite score. Higher NCD-Protect scores indicate consumption of a greater number of protective food groups.

The NCD-Risk score reflects consumption of food groups associated with dietary risk factors for non-communicable diseases, including foods high in free sugars, salt, unhealthy fats, and processed meats. One point was assigned for consumption of each risk food group during the previous 24 h, and points were summed to generate the composite score. Higher NCD-Risk scores indicate consumption of a greater number of food groups associated with dietary risks.

Additional modules examined school food practices (breakfast, lunch, canteen use, pocket money, outside food purchases), exposure to food marketing, and the influence of media, peers, and celebrities, all rated on a 5-point Likert scale (1 = least; 5 = most influential).

Enumerators received two days of training on ethics and interviewing methods, followed by simulations and supervised fieldwork. The questionnaire was pretested with 20 out-of-sample mothers, resulting in minor revisions for clarity and cultural relevance. Final interviews were conducted privately on school grounds.

### Qualitative Data Collection

Two focus group discussions (FGDs) were conducted with purposively selected mothers/caregivers from one slum and one non-slum community. Participants were recruited from mothers/caregivers who had completed the quantitative survey and expressed willingness to participate in a follow-up discussion. Each FGD included up to ten participants. Discussions explored perceptions of healthy diets, affordability and accessibility of nutritious foods, household food purchasing and preparation practices, barriers to providing healthy meals, and strategies for promoting healthy eating among children.

FGDs were conducted in Bangla by trained facilitators using a semi-structured discussion guide. Sessions were audio-recorded with participants’ permission, transcribed verbatim, and translated into English for analysis. Confidentiality was maintained throughout the qualitative data collection process.

### Data Analysis

Quantitative data were analyzed using SPSS version 20. Descriptive statistics were used to summarize household characteristics, food access, affordability indicators, and dietary outcomes. Differences between slum and non-slum groups were examined using Chi-square tests for categorical variables and Mann–Whitney *U* tests for continuous variables that did not meet normality assumptions.

Multivariable linear regression models were fitted to examine factors associated with children’s dietary outcomes. Primary models assessed predictors of Dietary Diversity Score (DDS) and weekly fruit and vegetable intake. Independent variables were selected a priori based on conceptual relevance and previous literature, including household characteristics (housing status and household head education), maternal characteristics (maternal education and employment status), and food environment indicators (frequency of vegetable purchasing, frequency of fruit purchasing, household food expenditure, and expenditure on fruits and vegetables). The same set of covariates was included across models to facilitate comparison of determinants across dietary outcomes. Additional adjusted regression models examined associations between household–maternal characteristic profiles and NCD-related dietary indicators, including NCD-Protect and NCD-Risk scores. Residence status was examined together with maternal education and maternal occupation to assess differences in dietary outcomes across combined household and maternal characteristic groups. Continuous expenditure variables were log-transformed to reduce skewness. Model assumptions, including linearity, residual distribution, and multicollinearity, were assessed before interpretation. Regression coefficients (β), standard errors (SE), and *p*-values are reported.

Qualitative data were analyzed using a framework approach. Initial coding focused on domains relevant to interpretation of the quantitative findings, including perceptions of healthy diets, affordability, food preferences, and household food practices. Themes were developed through iterative review of coded transcripts and were compared with quantitative findings to provide explanations for observed differences between slum and non-slum households.

## Results

### Participant, Household, and Socioeconomic Characteristics

Among the 300 participating children, 56% were from slum households and 44% from non-slum households. Children were within the targeted age range of 8–12 years, with comparable age distributions between slum and non-slum groups. The proportion of female children was slightly higher in slum schools than non-slum schools (57.5% vs. 41.4%, *p* = 0.008).

Household characteristics differed substantially by residence. Households in slum areas had larger family sizes (mean 4.69 vs. 4.32, *p* = 0.006), with 65% having more than four members compared to 51% in non-slum areas (*p* = 0.013). Disparities in education were also evident, with a higher proportion of household heads in slum areas having no formal education compared with non-slum areas (29% vs. 8%, *p* < 0.001). The median annual per capita income was BDT 60,000 (≈USD 484; 1 USD = BDT 124) in slum areas and BDT 105,000 (≈USD 847) in non-slum areas (*p* < 0.001). Slum households also had smaller dwelling areas (median 350 vs. 650 sq. ft., *p* = 0.013) and poorer housing facilities; only 56% had private kitchen, bathroom, and toilet facilities compared with 82% of non-slum households, while shared access to all three facilities was more common in slum households (33% vs. 9%, *p* < 0.001) (Table 1).

**Table 1:** Socio-demographic and household characteristics of study participants by residence

Variable	Total (*N* = 300)	Non-slum (*n* = 133)	Slum (*n* = 167)	*p*-value
**Household socioeconomic characteristics**				
Household size (members), mean (SD)	4.48 (1.18)	4.32 (1.11)	4.69 (1.23)	0.006
Household income (BDT), median (IQR)	25,000 (18,000–40,000)	40,000 (30,000–60,000)	20,000 (15,000–28,000)	< 0.001
Dwelling area (sft), median (IQR)	500 (200–700)	650 (500–1000)	350 (120–550)	0.013
House ownership (rented), *n* (%)	209 (70%)	97 (73%)	112 (67%)	0.272
**Dwelling status, *n* (%)**				< 0.001
– Private kitchen, bathroom & toilet	203 (68%)	109 (82%)	94 (56%)	
– Shared 1–2 facilities	30 (10%)	12 (9%)	18 (11%)	
– Shared all 3 facilities	67 (22%)	12 (9%)	55 (33%)	
**Household head characteristics**				
Age (years), median (IQR)	40 (35–45)	42 (38–45)	38 (35–42)	0.032
Female-headed household, *n* (%)	19 (6%)	8 (6%)	11 (7%)	0.84
**Education level, *n* (%)**				< 0.001
– No education	59 (20%)	11 (8%)	48 (29%)	
– SSC/HSC	172 (57%)	71 (53%)	101 (60%)	
– Hons & above	69 (23%)	51 (38%)	18 (11%)	
**Maternal characteristics**				
Age (years), median (IQR)	33 (30–37)	35 (30–39)	32 (29–36)	0.041
**Education level, *n* (%)**				< 0.001
– No education	54 (18%)	11 (8%)	43 (27%)	
– Educated	246 (82%)	122 (92%)	124 (74%)	
Employment (housewife), *n* (%)	219 (73%)	100 (75%)	119 (71%)	0.421

### Children’s Diet Quality

Children’s diets showed limited diversity and suboptimal fruit and vegetable intake. Overall, only 40% of children consumed all five recommended food groups in the previous 24 h, and the mean daily intake of fruits and vegetables was 3.7 servings, below the global recommendation of five servings per day.

Clear disparities were evident by residence and maternal education. Based on the seven-day food frequency questionnaire (SQFFQ), children in non-slum areas consumed significantly more fruits and vegetables than those in slum areas (4.7 vs. 3.0 servings/day, *p* < 0.001), and a higher percentage consumed all five recommended food groups during the previous 24 h (48% vs. 33%, *p* = 0.010). Maternal education also played a key role: children of mothers with formal education consumed 4.1 servings of fruits and vegetables per day, compared with 2.3 servings among children of mothers without formal education (*p* < 0.001). Based on the 24-h recall (DQQ), fruit consumption was higher among children in non-slum areas than those in slum areas (70% vs. 50%, *p* = 0.001). Animal-source foods were widely consumed, with approximately 90% of children reporting consumption during the previous 24 h. Consistent with these patterns, a weak but significant positive correlation was observed between household income and children’s dietary diversity (*r* = 0.12, *p* = 0.04), suggesting that children from higher-income households (≥ BDT 43,839 per month; ≈ USD 354) were more likely to have consumed all five food groups during the previous 24 h.

Dietary diversity scores were significantly higher among children living in non-slum areas (6.05 vs. 5.35, *p* < 0.001), among those whose mothers had formal education (5.85 vs. 4.83, *p* < 0.001), and among those whose mothers were housewives (5.83 vs. 5.23, *p* = 0.012) (Table 2). NCD-Protect scores were significantly higher among children of educated mothers and housewives but did not differ by residence status. Conversely, children living in non-slum areas had significantly higher NCD-Risk scores than those in slum areas (2.98 vs. 2.68, *p* = 0.044).

**Table 2:** Dietary diversity and NCD-related scores by household and maternal characteristics

Category	DDS (mean)	*p*-value	NCD-Protect (mean)	*p*-value	NCD-Risk (mean)	*p*-value
All children	5.67	–	3.87	–	2.82	–
**Residence status**						
Non-slum	6.05	< 0.001	4.00	0.587	2.98	0.044
Slum	5.35		3.71		2.68	
**Maternal education**						
Educated	5.85	< 0.001	3.99	0.017	2.85	0.412
No education	4.83		3.30		2.67	
**Maternal occupation**						
Housewife	5.83	0.012	4.00	0.032	2.77	0.781
Employed/other	5.23		3.52		2.93	

However, average intakes masked substantial gaps in adequacy. Based on the 24-h recall (DQQ), 11.3% of children consumed no vegetables and 41.0% consumed no fruits during the previous day. Seven-day frequency data from the SQFFQ showed similar patterns, although complete non-consumption over an entire week was less common: 2.0% of children reported no vegetable intake and 11.0% reported no fruit intake during the previous seven days. No fruit consumption during the previous week was more common among children in slum areas than among those in non-slum areas (16.7% vs. 5.3%). These findings indicate both short-term and habitual deficiencies in fruit and vegetable intake, particularly among children living in slum areas, with the disparity being especially pronounced for fruit consumption.

### Children’s Eating Behavior on a Typical School Day

Patterns of school-day eating behaviors showed clear contrasts between children living in slum and non-slum areas. On a typical school day, children in non-slum areas and those with more educated mothers were more likely to bring food from home, whereas children living in slum areas more often purchased food outside school. Daily breakfast before school was more common among children living in non-slum areas (65% vs. 53%, *p* = 0.013) and among children of mothers with formal education (63% vs. 38%, *p* < 0.001). Similarly, more children living in non-slum areas carried lunch boxes (*tiffin*, a South Asian term for packed meal) from home (51% vs. 29%, *p* = 0.001), containing a higher percentage of homemade items (15% vs. 3.6%, *p* < 0.001). Children of mothers with formal education also more frequently carried a daily tiffin (44% vs. 13%, *p* < 0.001). Conversely, more children in slum areas received money from their parents to purchase food (61% vs. 36%) and received a higher average weekly amount (BDT 52.43 vs. BDT 38.65).

Among home-prepared tiffin items, noodles/pasta (107 reports), bread/flatbread-based items (104 reports), and rice-based meals (76 reports) were most frequently reported. Protein-containing items were also commonly included, including eggs (63 reports) and animal-source foods such as fish, meat, chicken, and related items (42 reports). However, vegetables were rarely reported as part of home-prepared tiffin (18 reports). In comparison, purchased tiffin items were largely composed of bakery products (183 reports) and fried/savory snack foods (40 reports), while fast foods, sweets, and sugar-sweetened beverages were less frequently reported (Table 3). These findings suggest that although home-prepared tiffin was not consistently rich in fruits and vegetables, it provided greater opportunity for inclusion of protein-rich foods and meals prepared under household control, compared with food purchased outside school.

**Table 3:** Types of food items consumed as school tiffin by residence

Tiffin source	Food category	Examples of reported items	Non-slum *n*	Slum *n*	Total *n*
**Home-prepared**	Rice-based meals	Biriyani, fried rice, khichuri, rice	45	31	76
	Bread/flatbread-based items	Bread, chapati, pancake, paratha	47	57	104
	Egg-based items	Egg	31	32	63
	Noodles and pasta	Noodles, pasta	52	55	107
	Animal-source meals/snacks	Burger, chicken fry, fish, meat, nuggets	36	6	42
	Vegetable-based dishes	Mixed vegetables	11	7	18
	Savoury snacks	Samucha, sandwich, singara, vegetable roll	8	6	14
	Other	Other reported homemade foods	6	1	7
	**Total reported home-prepared items**		**236**	**195**	**431**
**Purchased outside food**	Bread/bakery-based items	Cake, biscuit, butter bun, bread, chapati, paratha	89	94	183
	Fried/savoury snacks	Chips, chanachur, popcorn, singara, samucha, fuchka, bhelpuri, chotpoti	23	17	40
	Fast food items	Burger, pizza, hot dog, chicken ball	8	1	9
	Sweets and desserts	Chocolate, jelly, ice cream, jilapi	5	7	12
	Sugar-sweetened beverages	Juice, soft drinks	1	3	4
	**Total reported purchased items**		**126**	**122**	**248**

Multiple food items could be reported for the same child; therefore, totals represent reported food items and are not mutually exclusive categories

### Children’s Exposure to Digital and Traditional Media

Exposure to digital and traditional media differed by residence (Table 4). Non-slum children had greater access to the Internet (90% vs. 68%, *p* < 0.001) and reported more time spent online (7 vs. 4 h/week, *p* = 0.001) compared with slum children. Internet access was also higher among children of mothers with formal education and among children of housewives, although time spent online did not differ significantly by maternal occupation. Most children accessed the Internet using their parents’ mobile phones. Perceived sources of influence on children’s food preferences also varied by residence, with non-slum children reporting greater influence from digital and commercial media channels, particularly online platforms and television advertisements, while slum children reported greater perceived influence from food packaging, celebrity endorsements, and interpersonal or community-based sources (Table 4).

**Table 4:** Food environment factors influencing children’s food preferences by residence

**Food environment factor**	**Average score (1–5)**		***p*-value**
	**Non-slum**	**Slum**	
Online platforms (websites, social media, etc.)	3.83	3.29	< 0.001
Television advertisements	3.51	3.00	0.001
Food packaging and labels	3.53	4.10	0.004
Peer recommendations	3.43	3.01	0.004
Celebrity endorsements	2.89	3.58	0.001
Family members or relatives	2.86	3.28	0.012
Educational programs or school curricula	2.56	3.06	0.003
Personal experiences or anecdotes	2.47	3.21	< 0.001
Health professionals (doctors, nutritionists)	2.33	3.16	< 0.001
Local community events or initiatives	2.26	3.04	< 0.001

Scores indicate perceived influence on food preferences (1 = not influential, 5 = very influential)

### Household Food Environment

#### Market Access and Availability

Households in both slum and non-slum neighborhoods relied heavily on local shops and street vendors for food acquisition (Supplementary Table  S1). However, slum households were substantially more likely to obtain foods from street vendors, particularly meat and fish (63% vs. 37%), eggs and dairy products (50% vs. 19%), and staple foods such as rice, wheat, and oil (47% vs. 12%) (all *p* < 0.001). In contrast, sources of vegetables and fruits were broadly similar between groups, although non-slum households were more likely to obtain fruits from village sources (24% vs. 15%, *p* = 0.049).

Fresh produce markets were more accessible to households in non-slum neighborhoods: 76% of respondents reported being within a 15-min walk compared to 69% for households in slum neighborhoods (*p* = 0.003). This disparity contributed to notable differences in fruit purchasing. In the past month, 27% of households in slum areas reported not buying any fruits, compared to 7% in non-slum areas (*p* < 0.001). Households in non-slum areas purchased fruits more frequently (4.19 times/month) than those in slum areas (1.98 times/month), a pattern strongly linked to household income (*r* = 0.446, *p* < 0.01). Vegetable purchasing frequency was similar between groups and showed no significant association with income.

Differences were also seen in the variety of produce available at home (Table 5). The median number of vegetable types available was higher in non-slum households than slum households (4 vs. 3 types, *p* < 0.001), while the median fruit variety was substantially higher in non-slum households (3 vs. 1 type, *p* < 0.001). Among non-slum households, 43.6% reported having 5–9 different vegetable types available in the previous week, compared with 24.6% of slum households (*p* < 0.001). Conversely, 12.0% of slum households reported no vegetables available at home, compared with none in non-slum households (*p* < 0.001). Fruit availability showed a similar pattern, with 35.3% of slum households reporting no fruits compared with 4.5% of non-slum households (*p* < 0.001).

**Table 5:** Diversity of vegetables and fruits available in households during the previous 7 days by residence

Food group	Number of types available	Non-slum (%)	Slum (%)	*p*-value
**Vegetables**	0	0.0	12.0	< 0.001
	1–4	56.4	63.5	
	5–9	43.6	24.6	
	**Median (IQR)**	**4 (3–6)**	**3 (2–4)**	** < 0.001**
**Fruits**	0	4.5	35.3	< 0.001
	1–3	66.9	58.7	
	4–7	28.6	6.0	
	**Median (IQR)**	**3 (2–4)**	**1 (0–2)**	** < 0.001**

Number of types available refers to the count of distinct vegetable or fruit items reported to be available in the household during the previous 7 days

#### Affordability

Income and education strongly influence food expenditure, and households in slums face greater financial constraints in accessing adequate food, particularly fruit and vegetables. Households in slum areas spent less on food (BDT 10,446 ≈ USD 84.24 vs. BDT 16,751.88 ≈ USD 135.10/month, *p* < 0.001) but allocated a higher percentage of their income to food (48.5% vs 35.7%, *p* < 0.001), indicating a higher financial burden on these households. Food expenditure was strongly and positively correlated with household income (*r* = 0.618, *p* < 0.001) and mother’s income (*r* = 0.614, *p* < 0.001), and moderately correlated with the age of the children household head (*r* = 0.377, *p* < 0.001). Households with a head who attained higher education spent more on food (29% spent > BDT 20,000 ≈ USD 161.29) compared to those with non-educated heads (1.7%, *p* < 0.001). The average expenditure on vegetables and fruits in non-slum areas was BDT 5512.8 ≈USD 44.46 per month in comparison to BDT 3107.8 ≈USD 25.06 in slum areas (*p* < 0.001). Fruit and vegetable expenditure has a strong and positive correlation with total food expenditure (*r* = 0.744, *p* < 0.001), household income (*r* = 0.401, *p* < 0.001), and mother’s income (*r* = 0.371, *p* = 0.001).

Most households, both in slum and non-slum neighborhoods, reported an inability to buy enough vegetables. For fruits, 98% of households in slums and 80% of households in non-slums could not purchase enough (*p* < 0.001). Food insecurity highlights economic disparities: 28% of households in slums often change their daily food choices due to financial constraints, compared to only 4% in non-slum areas (*p* < 0.001). This notable difference emphasizes the greater economic hardships and limited food access faced by those in slum areas.

### Predictors of Diet Quality and Fruit and Vegetable Intake

After accounting for household socioeconomic and maternal characteristics, fruit purchasing frequency and monthly fruit and vegetable expenditure were the only factors significantly associated with both children’s dietary diversity score (DQQ) and weekly fruit and vegetable intake (Table 6). Specifically, children from households purchasing fruit at least once weekly had a 0.91-point higher DQQ score (*p* < 0.001) and consumed approximately 9 additional weekly servings of fruits and vegetables (95% CI 0.76–16.04; *p* = 0.026) compared with those from households that did not purchase fruit. Similarly, children from households with higher monthly spending on fruits and vegetables had a 0.60-point higher DQQ score (*p* = 0.005) and approximately 11.7 additional weekly servings of fruits and vegetables (95% CI 3.29–19.51; *p* = 0.005). Household head education was also associated with weekly fruit and vegetable intake, with children from households headed by an educated individual consuming 10.6 more weekly servings than those whose household head had no formal education (*p* = 0.046). All other household socioeconomic and maternal characteristics were not significantly associated with either outcome after adjustment. Notably, residence (slum vs. non-slum) and maternal education, which were significant descriptively (Table 2), were no longer significant in the adjusted models. These findings suggest that behavioral and financial access to fruits and vegetables, rather than broad socioeconomic status alone, are the most immediate determinants of children’s dietary quality.

**Table 6:** Adjusted mean *differences* in children’s dietary diversity score and weekly fruit and vegetable intake from multivariable linear regression models (*N* = 300)

Predictor	Dietary diversity score (0–10)		Weekly servings of fruit and vegetables	
	Adjusted mean difference (SE)	*p*-value	Adjusted mean difference (SE)	*p*-value
Household characteristics				
Residence status	−0.084 (0.221)	0.704	−2.560 (4.342)	0.556
(ref = slum) (*n* = 168)				
Own house	− 0.282 (0.208)	0.178	+ 0.683 (4.087)	0.867
(ref = rented) (*n* = 91)				
Head educated	+ 0.183 (0.270)	0.499	+ 10.611 (5.297)	0.046
(ref = no education) (*n* = 59)				
Maternal characteristics				
Mother educated	− 0.461 (0.280)	0.101	− 0.437 (5.494)	0.937
(ref = no education) (*n* = 55)				
Mother employed	− 0.280 (0.229)	0.222	+ 5.870 (4.494)	0.193
(ref = unemployed) (*n* = 218)				
Household food purchasing and spending				
Vegetable purchasing ≥ 4 times per week (ref < 4 times) (*n* = 124)	+ 0.045 (0.196)	0.820	− 0.456 (3.850)	0.906
Fruit purchasing ≥ 1 × per week (ref = none) (*n* = 177)	+ 0.910 (0.204)	0.000	+ 8.990 (4.011)	0.026
Monthly food expenditure (log) (*n* = 300)	− 0.078 (0.249)	0.754	+ 4.974 (4.884)	0.309
Monthly fruit and vegetable spending (log) (*n* = 300)	+ 0.597 (0.212)	0.005	+ 11.703 (4.158)	0.005

Values are adjusted mean differences with standard errors from multivariable linear regression models. Positive values indicate higher dietary diversity or higher fruit and vegetable intake compared to the reference category. Food expenditure variables are log-transformed; coefficients represent change per unit increase in log expenditure (approximately proportional increase in spending)

### Mothers’ Perspectives

Mothers’ accounts during the FGDs contextualized observed quantitative patterns in children’s diets. Four interconnected themes emerged from the framework analysis: perceptions of healthy diets, affordability constraints, children’s food preferences, and parental feeding practices (Table 7). Lower dietary diversity among children living in slum areas reflected maternal focus on animal-source foods over fruits and vegetables, whereas mothers in non-slum areas emphasized diversity and balance in defining “healthy” meals. Vegetable aversion was common, with mothers in non-slum areas using active strategies to encourage their children to eat more vegetables, while mothers in slum areas prioritized meat to satisfy children’s preferences. While fruits were generally viewed as expensive, mothers in non-slum neighborhoods were more willing to purchase them when affordable or in season, whereas mothers in slum neighborhoods regarded them as occasional foods.

**Table 7:** Mothers’ perspectives explaining observed disparities in children’s diet quality, affordability, and food choices

Quantitative pattern	Qualitative explanation (FGD interpretation)	Selected representative quotes
Different physical access to food	Mothers from both settings reported that food was generally available, but the nature and convenience of access differed. Slum areas depended on nearby wholesale suppliers offering affordable options, while non-slum areas benefited from greater diversity and convenience, including supermarkets, online shopping, and home delivery	**Slum**: “There are some wholesale markets near the slum so all types of food are available there.” (S003) **Non-slum:** “We do shopping and buy grocery item online. I can buy fish in five minutes.” (N004) “There are various shopping malls like Agora, Swapna, these are close to our door.” (N005) “The market is nearby, even vans come in front of the house.” (N007)
Lower dietary quality in slum areas	Mothers in slum areas perceive animal-source foods as “healthy,” while vegetables are considered supplementary or optional. Mothers in non-slum areas tended to define “healthy food” in terms of diversity and balance, including both plant- and animal-source foods	**Slum**: “Fish, meat, eggs, milk are healthy foods.” (S001) **Non-slum**: “Healthy food refers to vegetables, eggs, milk, small fish, and fruits.” (N002) “Fruits in season, rice, fish, meat, pulses, vegetables are usually healthy foods.” (N002)
Children’s vegetable aversion and parental strategies	Children’s aversion to vegetables was universal, but mothers in non-slum areas applied more active feeding strategies, while those in slum areas prioritized meat	**Slum**: “There are chickens two days a week, beef once a week, because kids like it.” (S002) “If it is meat, my child eats properly and says nothing else.” (S003) **Non-slum**: “If they don’t want to eat vegetables, give them vegetables with fish, meat.” (N001)
Particularly low fruit intake	Mothers in both groups viewed fruits as expensive. Mothers in slums tend to perceive it as occasional foods, while mothers in non-slums described a more adaptive approach, by buying seasonal or local fruits that are more affordable	**Slum**: “Not many can afford to keep that fruit every day because of high prices.” (S004, group response) “Eating fruit once a week is enough.” (S002) **Non-slum**: “Buying imported fruits is very expensive… we can feed the seasonal or local fruits that we can afford.” (N007)

These narratives highlight that while physical access to food was broadly adequate, economic and cognitive access, what mothers perceived as affordable and “healthy,” largely determined dietary quality. Mothers in slum areas prioritized energy-rich and animal-source foods, reflecting pragmatic responses to cost and preference constraints. In contrast, mothers in non-slum areas emphasized balance and diversity, driven by their need for convenience, higher purchasing power, and exposure to health information.

## Discussion

Children living in Dhaka’s urban slums face a combination of socioeconomic, environmental, and behavioral constraints to eating a healthy diet with sufficient amounts of fruit and vegetables. This study identifies clear disparities in dietary diversity, food sourcing, and exposure to nutrition risks between children in slum and non-slum settings. While some eating behaviors—such as skipping breakfast—are present across groups, children in slum areas exhibit significantly lower dietary diversity, reduced fruit and vegetable consumption, and a greater reliance on foods from informal markets. These differences reflect household choices but also structural inequalities.

### Socioeconomic and Cognitive Inequities: Foundational but Indirect Drivers

Income and education disparities were evident between slum and non-slum areas, with households in slum areas earning nearly half as much per month. Households in slum areas face greater financial strain, allocating a larger share of their income to food, which limits their ability to afford diverse diets and results in lower expenditure on fruit and vegetables. While this finding aligns with global literature identifying affordability as a primary barrier to healthy eating in urban low-income populations [32], our qualitative data suggest that economic constraints also shape how mothers define and prioritize “healthy” foods for their children.

Mothers in slum areas associate healthfulness with filling, protein-rich foods that are viewed as strengthening and aspirational. In contrast, mothers in non-slum areas focus on diversity and balance, indicating greater nutrition literacy. These differing perceptions highlight that nutritional inequality is not only financial but also cognitive, rooted in varying knowledge and aspirations shaped by social class and education. The preference for animal-source foods among mothers in slums also reflects an aspirational aspect of Bennett’s Law, where higher-status foods like meat, fish, and eggs are linked with strength, health, and social mobility [33]. These perceptions influence dietary choices directly. In slum settings, children’s preference for animal-source foods reinforces maternal focus on satiety rather than variety. Meanwhile, mothers in non-slum areas actively employ strategies to promote vegetable consumption. Similarly, although both groups identified fruit affordability as a challenge, their responses differed. Mothers in non-slum areas adjusted their purchases based on seasonality and affordability, while mothers in slum areas saw fruits as optional and discretionary. This indicates that affordability interacts with perceptions of value, with mothers in slums prioritizing satiety, whereas other mothers see fruits as investments in health. Policy efforts focusing only on prices may therefore fail without also addressing the social and cognitive perceptions of diet.

Maternal occupation showed an association with dietary outcomes in unadjusted analyses, with children of non-employed mothers exhibiting higher dietary diversity and NCD-protect scores. However, this association was attenuated after adjustment for residence and maternal education, suggesting that maternal employment does not independently determine children’s diets but is instead embedded within broader socioeconomic conditions. Its influence likely operates through time availability, income stability, and access to food preparation resources, which vary across household contexts: mothers in more advantaged households may have access to greater financial resources, childcare support, and convenient food options, while mothers in low-income households may experience competing demands between income generation, time availability, and food preparation. Conversely, being a housewife may allow greater time for meal preparation but does not necessarily overcome constraints related to household income, food affordability, and access to diverse foods. These findings highlight that interventions should move beyond viewing maternal employment status as a direct determinant of child nutrition and instead address the structural conditions that influence caregivers’ capacity to provide healthy and diverse diets regardless of their employment status.

### Market Access and Food Environments: Availability versus Quality

While both groups described food as “available,” the quality and ease of access varied greatly. Mothers in non-slum areas mentioned supermarkets, shopping malls, and online grocery platforms as markets offering choice, convenience, and exposure to branded and processed foods. Households in slum areas relied on informal local markets and small vendors, often nearby, that usually sell affordable staples but have limited variety [34]. High transportation costs, time constraints, and limited home storage further hinder the purchase of perishable foods. Crowded living spaces and shared kitchens add to these challenges, making it harder to prepare fresh food regularly.

This distinction highlights that “access” is not merely spatial: households in non-slum areas enjoy greater access to market diversity, while those in slums experience market proximity without quality choice. These patterns reinforce research on the “informal market premium,” where low-income or informal neighborhoods often pay more for fresh fruit and vegetables even when vendors are physically close. For example, in Dhaka’s slums, mobile vendors charge more for vegetables than fixed markets [35]. Similar patterns have been observed in low-income neighborhoods in the United States [36].

### Children’s Food Behaviors and the School Food Environment

Socioeconomic gradients extended into children’s school-day food routines. Breakfast skipping was more prevalent among children in slums, reflecting both economic constraints, limited maternal education and caregiving capacity, consistent with previous studies [37, 38]. Children in non-slum areas were more likely to bring homemade tiffin, whereas children in slums were more likely to receive cash from their parents to buy food, which exposed them to unhealthy food choices. This pattern situates schools as both risk and opportunity points for improving children’s dietary environments.

Home-prepared tiffin is generally perceived as a healthier option because it allowed greater control over ingredients, hygiene, and cooking practices. Compared with purchased foods, home-prepared items more frequently included protein-rich foods such as eggs, fish, and meat, suggesting greater potential for balanced meals. However, fruits and vegetables were still uncommon in both settings, indicating that home preparation alone does not guarantee a high-quality diet. Strengthening school food policies, promoting healthier home-prepared meals, and improving the nutritional quality of available school food options may help improve children’s daily food choices [39–42].

### Media and Marketing Influences on Dietary Preferences

Media exposure shapes children’s dietary aspirations in distinct ways across socioeconomic groups. Children in non-slum areas, with greater Internet access, are more influenced by digital marketing and online food imagery, while packaging, celebrity endorsements, and interpersonal communication influence children in slums. Mothers in slum neighborhoods also cited community programs, health professionals, and teachers as trusted information sources—an entry point for targeted public nutrition campaigns.

Consistent with global evidence, cumulative exposure to unhealthy food advertising is positively associated with snack consumption and poor dietary outcomes [25]. Unregulated marketing of ultraprocessed foods contributes to the double burden of malnutrition in urban contexts [43]. Equitable media regulation and child-focused nutrition messaging, tailored to each group’s exposure patterns, are thus essential.

### Policy and Research Implications

Collectively, these findings emphasize that dietary inequities among urban children are co-produced by structural and behavioral constraints. Policies that focus solely on nutrition education or individual choice will fail without parallel measures to enhance affordable, diverse, and safe food access in slum neighborhoods. Addressing the significant financial burden on households in slum areas, through targeted food assistance or subsidies that include perishable and protective foods and not just staple grains, could directly improve children’s dietary diversity. Such efforts should be complemented by investments in infrastructure for safe storage, upgrading informal markets, and stronger integration of local food systems. This is consistent with evidence that modifiable parental behaviors, home and neighborhood environments jointly influence children’s dietary habits beyond physical access to healthy food [44].

At the same time, interventions must recognize that dietary aspirations differ by class. Nutrition programs should explicitly address the social meaning of “healthy” food, emphasizing that vegetables and fruits are not merely side dishes but essential for growth and protection. Community-based participatory programs that empower mothers to reinterpret these norms may be particularly effective.

At the school level, establishing standardized meal policies, regulating food vendors near schools, and integrating nutrition education can strengthen healthy eating habits. Promoting home-cooked meals and educating parents about healthy lunch options are also essential. Some existing programs in India, Brazil, and Thailand offer valuable examples that demonstrate well-designed school food policies can greatly enhance dietary diversity and decrease exposure to ultraprocessed foods [45–47].

While children in non-slum areas generally had more balanced diets, reflected in higher NCD-Protect scores, they also exhibited higher NCD-Risk scores. This suggests that urban dietary transitions are creating new risks alongside old ones, as greater food choice and purchasing power increase exposure to calorie-dense or processed foods. Understanding these diverging dietary pathways underscores the need for comprehensive, equity-oriented urban nutrition policies that simultaneously address undernutrition, overnutrition, and food environment inequities.

## Conclusion

Dietary inequities among urban children in Dhaka are co-produced by structural barriers and household behaviors. Lower dietary diversity among children in slum neighborhoods stems from constrained household income, limited market access, and entrenched perceptions of “healthy” food that prioritize energy-rich and animal-source items over fruit and vegetables. These dynamics reveal that improving child nutrition requires more than changing individual preferences; it demands addressing the systemic conditions that restrict food choices.

Efforts to close this nutritional gap must therefore combine structural and behavioral strategies. Policies should extend beyond staple food subsidies to include perishable, nutrient-rich foods, supported by investments in storage, market infrastructure, and informal vendor upgrading. At the same time, participatory programs that engage parents and schools can help reshape norms around healthy eating and strengthen children’s daily food environments. Recognizing the dual burden of undernutrition in slums and rising NCD risks in non-slum areas, multi-sectoral, equity-focused interventions will be essential to achieving healthier urban diets for all children.

## Supplementary Information

Below is the link to the electronic supplementary material.

ESM 1 (DOCX 16.7 KB)

## Data Availability

The data are not publicly available to protect participant privacy, but anonymized data may be made available upon reasonable request.
