# Supplementary material for: Inequities in Children’s Food Environment: A Comparison between Slum and Non-slum Settings in Urban Bangladesh
Source: J Urban Health. 2026 Aug 31;103(5):955–70. doi: 10.1007/s11524-026-01144-5 (PMC13672271; doi:10.1007/s11524-026-01144-5)
Supplement: Supplementary file 1 [file 11524_2026_1144_MOESM1_ESM.docx]

## Supplementary Materials

Supplementary Table S1: Household-reported food acquisition sources (%) among slum and non-slum households

| Food group | Non-Slum (%) | Slum (%) | p-value |
| --- | --- | --- | --- |
| Meat & Fish: | | | |
| Cart | 37.2 | 63.4 | <0.001 |
| Local shop | 90.7 | 89.0 | 1.000 |
| Own cultivation | 1.6 | 3.0 | 0.469 |
| From village | 16.3 | 11.6 | 0.306 |
| Other | 4.7 | 4.2 | 0.257 |
| Vegetables: | | | |
| Cart | 87.8 | 89.6 | 0.729 |
| Local shop | 87.8 | 80.5 | 0.127 |
| Own cultivation | 6.9 | 3.0 | 0.169 |
| From village | 22.9 | 15.2 | 0.100 |
| Other | 1.6 | 2.4 | 1.000 |
| Fruits: | | | |
| Cart | 65.1 | 71.3 | 0.218 |
| Local shop | 85.3 | 77.4 | 0.199 |
| Own cultivation | 4.7 | 1.8 | 0.192 |
| From village | 24.0 | 14.6 | 0.049 |
| Other | 12.4 | 9.7 | 0.323 |
| Egg, milk & dairy products: | | | |
| Cart | 19.2 | 49.7 | <0.001 |
| Local shop | 97.7 | 89.7 | 0.036 |
| Own cultivation | 3.8 | 1.8 | 0.473 |
| From village | 15.4 | 13.3 | 0.783 |
| Other | 5.4 | 9.1 | 0.313 |
| Rice, wheat, oil: | | | |
| Cart | 11.5 | 47.0 | <0.001 |
| Local shop | 93.8 | 93.9 | 1.000 |
| Own cultivation | 1.5 | 3.7 | 0.308 |
| From village | 8.5 | 9.1 | 1.000 |
| Other | 10.0 | 10.3 | 1.000 |

*Notes: Cart= street vendors or mobile vendors selling food items. Local shop= neighborhood stores or smaller retail outlets. Village= from village relatives or own cultivated land in village. Percentages represent the proportion of households reporting each source for obtaining the specified food group. Households could report multiple food acquisition sources; therefore, percentages within each food group may sum to more than 100%.*
